# Associations between Lipid Profiles and Graves’ Orbitopathy can Be HLA-Dependent

**DOI:** 10.3390/genes14061209

**Published:** 2023-05-31

**Authors:** Magdalena Stasiak, Katarzyna Zawadzka-Starczewska, Bogusław Tymoniuk, Bartłomiej Stasiak, Andrzej Lewiński

**Affiliations:** 1 Department of Endocrinology and Metabolic Diseases, Polish Mother’s Memorial Hospital—Research Institute, 281/289 Rzgowska St., 93-338 Lodz, Poland; mstasiak33@gmail.com (M.S.); katarzyna.zawadzka-starczewska@iczmp.edu.pl (K.Z.-S.); 2 Department of Immunology, Rheumatology and Allergy, Medical University of Lodz, 251 Pomorska St., 92-213 Lodz, Poland; boguslaw.tymoniuk@gmail.com; 3 Institute of Information Technology, Lodz University of Technology, 215 Wolczanska St., 90-924 Lodz, Poland; bartlomiej.stasiak@p.lodz.pl; 4 Department of Endocrinology and Metabolic Diseases, Medical University of Lodz, 281/289 Rzgowska St., 93-338 Lodz, Poland

**Keywords:** Graves’ orbitopathy, Graves’ disease, human leukocyte antigen, HLA, TSH receptor antibodies, risk factors, LDL cholesterol, total cholesterol

## Abstract

The risk of Graves’ orbitopathy (GO) is related to the human leukocyte antigen (HLA) profile and was demonstrated to be increased in patients with elevated total cholesterol (TC) and/or low-density lipoprotein (LDL) cholesterol. We hypothesized that there were some HLA alleles that were related to both GO and TC and/or LDL levels. Therefore, the aim of the study was to compare the TC/LDL results in patients in whom GO-related HLA alleles were present to those in whom they did not occur. HLA classes were genotyped using a next-generation sequencing method in 118 patients with Graves’ disease (GD), including 63 and 55 patients with and without GO, respectively. Lipid profiles were assessed at the time of the GD diagnosis. A significant correlation between the presence of GO high-risk alleles (HLA-B*37:01 and C*03:02) and higher TC/LDL levels was found. Additionally, the presence of alleles associated with non-GO GD (HLA-C*17:01 and B*08:01), as well as alleles in linkage disequilibrium with B*08:01 (i.e., HLA-DRB1*03:01 and DQB1*02:01), was correlated with lower TC levels. These results further confirm the significance of TC/LDL in the risk of GO development and provide evidence that associations between TC/LDL and GO can be HLA-dependent.

## 1. Introduction

Graves’ disease (GD) is an autoimmune thyroid disorder (AITD) triggered by several exogenous or endogenous factors in genetically predisposed individuals [1]. Genetic susceptibility to GD is linked to many genes, and recently, a significant association with human leukocyte antigens (HLA) has been presented for the Caucasian population. The alleles of high-risk GD were as follows: HLA-B*08:01, -B*39:06, -B*37:01, -C*07:01, -C*14:02, -C*03:02, -C*17:01, -DRB1*03:01, -DRB1*11:01, -DRB1*13:03, -DRB1*01:03, -DRB1*14:01, -DQB1*03:01, and DQB1*02:01 [1]. The disease results from an autoimmune response against the thyroid stimulating hormone (TSH) receptor (TSHR) and subsequent thyroid receptor autoantibodies (TRAb) production [2]. These antibodies may exhibit high affinity for eye tissues, which leads to Graves’ orbitopathy (GO) development. Additionally, an important role of the insulin-like growth factor-1 (IGF-1) receptor (IGF-1R) was demonstrated [3]. IGF-1R activation in orbital fibroblasts may result from the binding of stimulatory IGF-1R antibodies (IGF-1R-Ab) to IGF-1R. Alternatively, synergistic crosstalk between TSHR and IGF-1R after binding of stimulatory TRAb to TSHR may lead to IGF-1R activation [4]. GO is associated with significantly reduced quality of life (QoL) due to exophthalmos and further severe complications, including optic neuropathy and/or corneal breakdown, which are sight-threatening conditions [5]. Due to the health burden carried by GO, extensive research on both genetic and non-genetic risk factors has been conducted for the last few decades. Very recently, we have demonstrated for the first time that in Caucasians, GO is strongly related to HLA, with HLA-A*01:01, -A*32:01, -B*37:01, -B*39:01, -B*42:01, -C*08:02, C*03:02, DRB1*03:01, DRB1*14:01, and DQB1*02:01 being genetic markers of increased risk of GO, and *HLA*-C*04:01, -C*03:04, -C*07:02, and -DRB1*15:02 being protective alleles [6].

Among the non-genetic risk factors for GO, the importance of elevated total cholesterol (TC) and low-density lipoprotein (LDL) cholesterol levels has been demonstrated in recent years [5,7,8]. Therefore, a recommendation of active cholesterol-lowering therapy in patients with GD was introduced, especially in those with other high-risk factors such as smoking, high TRAb levels, or uncontrolled hyper- or hypothyroidism [3,5]. It should be emphasized that a recent report has demonstrated that the risk of GO is significantly increased not only in patients with high cholesterol levels but also with only a slight elevation of LDL and—which seems very important—with even high normal TC levels [9].

In developed countries, increased TC and/or LDL levels usually result from high saturated fat intake and a sedentary lifestyle. However, genetic background plays an important role in lipid synthesis and metabolism. Many genes were proven to be related to cholesterol metabolism, including, among others, the proprotein convertase subtilisin/kexin type 9 (*PCSK9*) gene, the LDL receptor (*LDLR*) gene, the lipoprotein lipase (*LPL*) gene, and the apolipoprotein B (*APOB*) gene [10,11]. The HLA impact on lipid parameters was unclear.

Taking into account the strength of association between GO and HLA, as well as GO and TC and/or LDL levels, we hypothesized that there were some HLA alleles that were related to both GO and TC and/or LDL levels. Therefore, the aim of the study was to compare the TC and LDL results in patients in whom GO-related HLA alleles were present to those in whom they did not occur. Such an analysis had never been performed before, so the results can significantly broaden knowledge on the importance of HLA in the correlation between GO and lipid profiles.

## 2. Materials and Methods

### 2.1. Patients

The study included 118 individuals who were diagnosed with GD in the Department of Endocrinology and Metabolic Diseases, Polish Mother’s Memorial Hospital—Research Institute, Lodz, Poland, as well as in the department-associated outpatient clinic. This group is composed of 63 and 55 patients with and without GO, respectively.

### 2.2. Inclusion Criteria

In the included patients, GD was diagnosed on the basis of standard criteria [12], i.e., hyperthyroidism, elevated TRAb level, as well as a typical ultrasound (US) pattern. GO was diagnosed on the basis of the presence of lid retraction, eye soft-tissue involvement (redness, swelling), and exophthalmos, according to the actual guidelines [5]. In our GO group, orbitopathy occurred in most cases several months after GD onset and more rarely at the moment of GD diagnosis. In order to confirm patients’ group affiliation, long-term follow-up (>3 years) of all patients was required. Each patient in whom GO symptoms occurred during the follow-up period was reclassified and included in the GO group. In all the included patients, lipid profile results performed at the time of GD diagnosis were required. Patients with any acute or chronic diseases or with medications that may have influenced their lipid profile were excluded from the study.

### 2.3. Laboratory Procedures

In all patients, blood samples were collected at the time of the GD diagnosis. Samples were collected after 14-h overnight fasting. Serum levels of TC and LDL were measured by the VITROS^®^ 4600 Chemistry System (Ortho Clinical Diagnostics, Raritan, NJ, USA).

### 2.4. HLA Genotyping

DNA was isolated from whole blood samples, which were collected in anticoagulant (EDTA)-containing tubes. *HLA-A, -B, -C, -DQB1,* and *-DRB1* were genotyped with a high-resolution next-generation sequencing (NGS) method with the application of the MIA FORA NGS FLEX 5 HT HLA Typing Kit [13] (Immucor Transplant Diagnostics, Inc., 35 Technology Drive, South Warren, NJ 07059, USA). The MIA FORA NGS FLEX 5 HT HLA typing protocol applies long-range PCR to capture the clinically relevant Class I and II HLA genes. The core kit includes each of the Class I genes, *HLA-A*, *HLA-B*, and *HLA-C*, as well as the Class II genes, *HLA-DRB1* and *HLA-DQB1*. *HLA-A*, *HLA-B*, and *HLA-C* are sequenced in their entirety. Sample preparations were divided into three distinct sections: long-range PCR, library preparation, and sequencing. During the first section, PCR mixes per sample were prepared. Every gene was amplified as one large piece in its entirety. The only exception was *DRB*, which was amplified as two overlapping segments because of its large size. Following gene amplification, amplicons were quantitated by fluorescence detection using PicoGreen™ reagent and a fluorescent plate reader. The sample PCR products were balanced and pooled before proceeding with enzymatic fragmentation, end repair, A-tailing, and cleaning with magnetic beads. Each kit contains two sets of six individual index adaptor plates, with 96 adaptors per plate. These index adaptors contain index sequences (barcodes) and Illumina-compatible adaptor sequences that allow for sequencing in a multiplex format. Index adaptors from identically named index adaptor plates (1–6) cannot be combined into the same library. Each 96-well sample plate was consolidated into a single microcentrifuge tube and size-selected with Pippin Prep before final PCR amplification. The library was quantitated by Qubit, and the concentration was adjusted according to the Illumina NextSeq library preparation protocol. This protocol describes semi-automated sample processing for high-throughput sequencing, from long-range PCR through library preparation, prior to sequencing on an Illumina instrument (Illumina, 5200 Illumina Way, San Diego, CA 92122, USA). The genomic library was cleaned with magnetic beads and denatured by 0.2N NaOH before loading on the NGS Illumina Platform. All automated sample processing was performed on the Biomek i7 Liquid Handler. Sequencing data were analyzed by MiaFora NGS software version 4.5 and the IPD-IMGT/HLA database version 3.40. The data were considered sufficient whenever the coverage reached 40. We used advanced NGS HLA Genotyping Software MIA FOR A, Sirona Genomics, Inc. Genotypes were computed from massive, paired-end sequencing reads derived from the Illumina Next-Generation Sequencing platform.

### 2.5. Statistical Analysis

Descriptive statistics of the collected data included the mean and standard deviation (SD). For comparisons between the groups, we used the Student’s *t*-test for normally distributed variables and the Mann-Whitney U test for the other ones. The normality of data distributions was assessed by the Shapiro-Wilk test. In all the tests, a *p* value < 0.05 was considered significant. Python statistical libraries (SciPy stats) were used for all the computations.

### 2.6. Ethics Procedures

Written informed consent for the performed procedures was obtained from all of the patients after a thorough explanation of their purpose and course. The study was approved by the Ethics Committee of the Polish Mother’s Memorial Hospital—Research Institute, Lodz, Poland (approval code—62/2020).

## 3. Results

The HLA alleles previously demonstrated to be associated with GO or non-GO GD, i.e., HLA-A*01:01, A*32:01, B*07:02, B*08:01, B*39:06, B*37:01, B*39:01, B*42:01, B*51:01, C*03:02, C*04:01, C*03:04, C*07:02, C*08:02, C*07:01, C*14:02, C*16:02, C*17:01, DRB1*03:01, DRB1*14:01, DRB1*15:02, and DQB1*02:01, DRB1*01:03, and DQB1*03:01 [6], were included in the analysis. On the basis of our previous report [6], the included alleles were divided into the following subgroups: (1) alleles associated with increased risk of GO (GO high-risk—GOHR), i.e., HLA-A*01:01, A*32:01, B*37:01, B*39:01, B*42:01, C*08:02, C*03:02, DRB1*03:01, DRB1*14:01, and DQB1*02:01; (2) alleles protective against GO (GO protective—GOP), i.e., C*04:01, C*03:04, C*07:02, and DRB1*15:02; (3) alleles associated with increased risk of non-GO, but not GO (non-GOHR), i.e., B*08:01, B*39:06, B*51:01, C*07:01, C*14:02, C*16:02, C*17:01, DRB1*15:02, and DQB1*03:01; allele DRB1*01:03 was excluded from statistical analysis as only one carrier of this allele was found in the analyzed group; and (4) alleles protective against non-GO (non-GOP), i.e., B*07:02, C*07:02, and A*32:01.

Statistically significant differences in the concentrations of TC and LDL were found for several alleles associated with GO/non-GO.

### 3.1. Association with GO High-Risk Alleles

Mean TC levels as well as mean LDL concentrations were significantly higher in patients with HLA-C*03:02 (Table 1) than in those without the presence of this allele. Total cholesterol levels were also significantly higher in carriers of HLA-B*37:01 as compared to allele-negative patients. The mean LDL concentrations were similarly higher, but the difference did not reach statistical significance (Table 1). In patients positive for either HLA-DRB1*03:01 or -DQB1*02:01, the mean TC level was significantly lower than in allele-negative patients. Such correlations were also present for mean LDL, but the differences were not statistically significant (Table 1). No other correlations between the GOHR allele and TC/LDL levels were found (Table 1).

### 3.2. Associations with GO Protective Alleles

No significant differences in mean TC/LDL levels were found between patients with and without any of the GOP alleles (Table 2).

### 3.3. Associations with Non-GO but Not GO High-Risk Alleles

Mean total cholesterol levels were significantly lower in carriers of HLA-B*08:01 and of HLA-C*17:01 as compared to allele-negative patients. Mean LDL concentrations were similarly lower in patients positive for any of the two alleles, but the difference did not reach statistical significance (Table 3).

### 3.4. Associations with Non-GO Protective Alleles

No significant differences in mean TC/LDL levels were found between patients with and without any of the non-GOP alleles (Table 4).

## 4. Discussion

The risk factors of GO have been thoroughly analyzed for the last few decades. However, HLA-related genetic predisposition was difficult to unequivocally confirm before the introduction of high-resolution NGS methods [6,14]. Many authors made attempts to find the HLA-associated background of GO, but the results were inconsistent due to low-resolution methods [15,16,17,18,19]. Recently, application of the NGS methods has finally led to the demonstration of correlations between HLA alleles and the risk of both GO and non-GO GD in the Caucasian population [6].

The significance of TC and LDL levels in GO development is also a novel finding [5,7,8,9]. No such relationship was demonstrated for other lipid parameters [8,9]. Concentrations of TC and LDL were positively correlated not only with GO risk but also with a GO CAS result [7,20]. Interestingly, TC and LDL levels were proven to have a strong impact on GO development even if they were only slightly elevated [6].

Such a significant correlation between GO and both HLA and TC and/or LDL should not be considered random. In the present study, we demonstrated that TC levels were significantly higher in carriers of two of the GO high-risk alleles, i.e., HLA-B*37:01 and HLA-C*03:02. In the case of the last allele, LDL concentrations were also significantly higher in carriers as compared to allele-negative patients. Interestingly, in patients with the other two alleles related to GO—HLA-DRB1*03:01 and DQB1*02:01—an inverse correlation was found, with a lower TC in carriers than in allele-negative patients. No relationship was found for the rest of the GO high-risk alleles. Finding the described opposite correlation seems to be of great value to demonstrate that some HLA alleles actually influence TC/LDL levels in GD patients. Both HLA-B*37:01 and -C*03:02 alleles belong to the major histocompatibility complex (MHC) class I, and the impact of MHC class I and class II on many pathological processes may be different [21]. The opposite influence of HLA-DRB1*03:01—an MHC class II allele—is therefore not an unexpected finding. However, there are also other potential reasons for this phenomenon. The HLA-related risk of GO was previously demonstrated to be much higher in carriers of HLA-B*37:01 and -C*03:02 as compared to HLA-DRB1*03:01 and -DQB1*02:01, with an odds ratio (OR) of 4.5 and 8.3 vs. 1.9 and 1.9, respectively [6]. Therefore, the strength of the allele’s impact on GO risk may be correlated with its influence on TC/LDL levels. On the other hand, HLA-DRB1*03:01 is an allele associated with many autoimmune diseases and is not specific to GO [22,23]. This lack of GO-specificity may further explain the current findings. There is a strong linkage disequilibrium between HLA-DRB1*03:01 and -DQB1*02:01 [24], so their similar impact found in our study should have been expected, and it further confirms the reliability of the results. On the other hand, there is no linkage disequilibrium between either HLA-B*37:01 or HLA-C*03:02 and any other of the GO-related alleles. Therefore, the impact of each of these two alleles should be considered entirely independent [25,26].

On the other hand, in the present study, we demonstrated that TC levels were significantly lower in carriers of two of the non-GO high-risk alleles, i.e., HLA-B*08:01 and HLA-C*17:01. These alleles were proven to be associated only with non-GO GD and not with GO risk. For none of the non-GOHR alleles, TC and LDL levels were higher in carriers as compared to non-carriers. As TC and LDL levels are higher in GO than in non-GO [9], the present finding can further confirm our hypothesis that some HLA alleles influence TC/LDL levels in GD. Similarly to the GOHR group, both alleles of potential significance belong to MHC class I. HLA-C*17:01 is not in linkage disequilibrium with HLA-B*08:01 or with any other non-GDHR alleles; therefore, its impact should be considered independent.

As linkage disequilibrium is related to the close proximity of a pair of loci along a chromosome [27], it is generally observed among alleles of the same MHC class. Interestingly, more frequent co-presence of HLA-B*08:01 with DRB1*03:01 and -DQB1*02:01 was observed [1,28,29], indicating possible linkage disequilibrium between these alleles, although they belong to different MHC classes. Our recent study demonstrated that in GD patients, HLA-B*08:01 was accompanied by -DRB1*03:01 and -DQB1*02:01 in most cases of its occurrence [1]. The co-presence of these three alleles in the GD group was four times more frequent than in controls. Moreover, HLA-DRB1*03:01 and -DQB1*02:01 were seldom present without HLA-B*08:01 [1]. Interestingly, in the present study, the impact of HLA-B*08:01 on TC levels was identical to that of HLA-DRB1*03:01 and -DQB1*02:01. This observation is consistent with the previous findings of possible linkage disequilibrium between these alleles and can further confirm our hypothesis of potential HLA-TC/LDL correlations in GD patients as well as the reliability of our current results. The same impact of alleles that are in linkage disequilibrium, despite the fact that they belong to different risk groups (GOHR vs. non-GOHR) on the TC level, additionally confirms the previously indicated existence of a specific mechanism of impact augmentation between these alleles in GD [1].

As this is the first report on a possible correlation between GO-related HLA alleles and TC/LDL levels, comparison with other authors’ results in GD was not possible. However, in patients with other HLA-associated diseases, relationships between lipid concentration and HLA profile have already been confirmed. In young patients with diabetes mellitus type 1 (DM1), HLA-DQ2/8 was strongly linked to an increased LDL-to-HDL ratio. This association remained significant after adjustment for age, glycosylated hemoglobin (HbA1c) level, and duration of DM1 [30].

*HLA-DRB1* alleles coding a five-amino acid sequence motif in residues 70–74 of the HLA-DRβ chain are called “shared epitopes” (SEs). This SE is associated with severe rheumatoid arthritis (RA) [31]. The main SE-coding alleles are composed of members of the HLA-DRB1*04 allele group (mainly DRB1*04:01, DRB1*04:04, DRB1*04:05, and DRB1*04:08), HLA-DRB1*01:01 or DRB1*01:02, HLA-DRB1*14:02, and HLA-DRB1*10:01 [30]. Toms et al. demonstrated that in patients with RA, *HLA-DRB1-SE* affects TC, LDL, and apolipoprotein B (ApoB) levels [32]. They reported a significant association between the presence of *HLA-DRB1-SE* and a higher TC-to-high-density lipoprotein (HDL) ratio, a higher ApoB-to-ApoA ratio, and lower ApoA levels [32].

The above-quoted studies confirmed the existence of correlations between HLA and lipid parameters in autoimmune diseases, as observed in our present study in a GD cohort. Rosacea is an inflammatory skin condition that shares HLA risk loci with such autoimmune diseases as DM1, celiac disease, multiple sclerosis, and/or RA [33]. Interestingly, the significance of HDL in mediation of the effect on the correlation between HLA-DQB1*03:03 and a risk of severity of rosacea was observed [34]. Therefore, the mechanism of the HLA effect on lipid parameters seems to be very complex, and the impact of HLA on several diseases may be modulated by lipid parameters. On the basis of our results, such a triggering effect of high TC and LDL levels on GO should also be taken into account in patients with particular GO high-risk alleles, i.e., *HLA-B37:01* and -C*03:02. On the other hand, a direct influence of HLA on lipids is supported by our finding of a lower level of TC in the GO group than in the non-GO group in carriers of such non-GOHR alleles/haplotypes as HLA-C*17:01 and B*08:01, DRB1*03:01, and DQB1*02:01. Therefore, HLA-lipid correlation is possibly a complex bidirectional phenomenon, and further studies are necessary to explore the mechanisms and significance of the presented findings, not only in GO and non-GO patients but also in other autoimmune conditions.

Relationships between HLA and lipid parameters were also studied in non-autoimmune conditions. A correlation between genetic variants of *HLA-DQB1* associated with human longevity and lipid parameters was reported by Yang et al. [35]. The authors identified four new SNPs in HLA-DQB1*05 (rs41542812), DQB1*03 (rs1049107, rs1049100), and DQB1*02 (rs3891176). The first three variants are in close linkage disequilibrium [35]. These HLA-DQB1 longevity-associated variants were related to better blood lipid profiles, including a lower LDL-to-HDL ratio [36]. Moreover, a beneficial effect of HLA-DR16 on the interactions between HDL and LDL with regard to LDL-related atherogenic processes was also found [36]. Therefore, correlations between HLA and lipid parameters were postulated in both autoimmune and non-autoimmune conditions.

Interestingly, in all the quoted studies, correlations were found for MHC class 2 alleles only, while in our study, the main correlations were found for MHC class 1. However, this observation cannot be considered a discrepancy because in the rest of the studies, only MHC class II alleles/antigens were analyzed, or only they were included in the correlation analysis due to their association with a given disease. Our study is the first in which the significance of HLA alleles in both MHC classes has been observed.

## 5. Conclusions

In the present study, a significant correlation was found between the presence of the GO high-risk alleles HLA-B*37:01 and HLA-C*03:02 and higher TC levels. Additionally, the presence of alleles associated with non-GO GD, i.e., *HLA C17:01* and B*08:01, as well as alleles in strong linkage disequilibrium with B*08:01, was correlated with lower TC levels. These results further confirm the significance of TC and LDL levels in the risk of GO development and provide evidence that associations between TC as well as LDL and GO can be HLA-dependent. Due to a potential bidirectional relationship between HLA and TC/LDL, our results are of great value as further indications for lipid-lowering treatment in GD patients, especially in those with GO high-risk alleles, particularly *HLA-B37:01* and *C03:02*. As our results are the first to demonstrate such associations in Caucasians, further studies are necessary to confirm these findings, especially because the determination of these associations can constitute an essential step in the development of personalized medicine focused on individual risk assessment.

## Figures and Tables

**Table 1:** Associations between TC and LDL levels and the presence of GO high-risk alleles.

HLA	Allele (+)	Allele (-)	*p* Value
	Mean TC ± SD (*n*)	Mean TC ± SD (*n*)	
HLA-A*01:01	168.41 ± 41.46 (44)	184.84 ± 58.44 (74)	0.15
HLA-A*32:01	197.40 ± 70.69 (5)	177.88 ± 52.52 (113)	0.60
HLA-B*37:01	232.40 ± 48.78 (5)	176.34 ± 52.28 (113)	0.03 *
HLA-B*39:01	201.67 ± 63.59 (6)	177.48 ± 52.60 (112)	0.37
HLA-C*03:02	224.29 ± 49.51 (7)	175.84 ± 52.27 (111)	0.02 *
HLA-C*08:02	211.20 ± 63.99 (5)	177.27 ± 52.51 (113)	0.22
HLA-DQB1*02:01	164.43 ± 40.70 (35)	184.74 ± 56.76 (83)	0.03 *
HLA-DRB1*03:01	162.51 ± 39.62 (35)	185.54 ± 56.77 (83)	0.01 *
HLA-DRB1*14:01	196.67 ± 52.32 (3)	178.24 ± 53.33 (115)	0.57
	**Mean LDL ± SD (*n*)**	**Mean LDL ± SD (*n*)**	
HLA-A*01:01	97.02 ± 33.22 (44)	112.43 ± 49.90 (74)	0.16
HLA-A*32:01	116.80 ± 51.46 (5)	106.24 ± 44.79 (113)	0.57
HLA-B*37:01	141.60 ± 43.63 (5)	105.14 ± 44.51 (113)	0.11
HLA-B*39:01	120.50 ± 52.38 (6)	105.95 ± 44.61 (112)	0.46
HLA-C*03:02	143.57 ± 47.98 (7)	104.36 ± 43.89 (111)	0.04 *
HLA-C*08:02	137.40 ± 47.15 (5)	105.33 ± 44.52 (113)	0.10
HLA-DQB1*02:01	95.46 ± 32.87 (35)	111.42 ± 48.49 (83)	0.13
HLA-DRB1*03:01	93.09 ± 31.41 (35)	112.42 ± 48.52 (83)	0.06
HLA-DRB1*14:01	119.67 ± 50.58 (3)	106.35 ± 44.94 (115)	0.54

* values statistically significant.

**Table 2:** Associations between TC and LDL levels and the presence of GO protective alleles.

HLA	Allele (+)	Allele (-)	*p* Value
	Mean TC ± SD (*n*)	Mean TC ± SD (*n*)	
HLA-C*03:04	144.25 ± 54.67 (4)	179.92 ± 52.95 (114)	0.16
HLA-C*04:01	173.10 ± 54.81 (20)	179.86 ± 53.04 (98)	0.68
HLA-C*07:02	166.60 ± 61.73 (10)	179.83 ± 52.49 (108)	0.36
HLA-DRB1*15:02	188.25 ± 65.65 (4)	178.38 ± 53.00 (114)	0.76
	**Mean LDL ± SD (*n*)**	**Mean LDL ± SD (*n*)**	
HLA-C*03:04	89.25 ± 47.30 (4)	107.30 ± 44.90 (114)	0.37
HLA-C*04:01	105.30 ± 48.07 (20)	106.97 ± 44.48 (98)	0.85
HLA-C*07:02	94.60 ± 49.27 (10)	107.81 ± 44.55 (108)	0.27
HLA-DRB1*15:02	114.25 ± 49.00 (4)	106.42 ± 44.96 (114)	0.62

**Table 3:** Associations between TC and LDL levels and the presence of non-GO high-risk alleles.

HLA	Allele (+)	Allele (-)	*p* Value
	Mean TC ± SD (*n*)	Mean TC ± SD (*n*)	
HLA-B*08:01	158.14 ± 32.15 (28)	185.11 ± 56.81 (90)	0.04 *
HLA-B*39:06	158.80 ± 67.39 (5)	179.59 ± 52.65 (113)	0.27
HLA-B*51:01	168.00 ± 51.29 (12)	179.93 ± 53.47 (106)	0.48
HLA-C*07:01	170.03 ± 41.75 (40)	183.17 ± 57.90 (78)	0.34
HLA-C*14:02	166.00 ± 56.34 (4)	179.16 ± 53.26 (114)	0.57
HLA-C*16:02	216.50 ± 103.94 (2)	178.06 ± 52.50 (116)	0.97
HLA-C*17:01	143.00 ± 31.30 (6)	180.62 ± 53.50 (112)	0.03 *
HLA-DQB1*03:01	183.61 ± 58.21 (54)	174.58 ± 48.59 (64)	0.37
HLA-DRB1*15:02	188.25 ± 65.65 (4)	178.38 ± 53.00 (114)	0.76
	**Mean LDL ± SD (*n*)**	**Mean LDL ± SD (*n*)**	
HLA-B*08:01	90.82 ± 27.49 (28)	111.62 ± 48.13 (90)	0.08
HLA-B*39:06	98.40 ± 47.51 (5)	107.05 ± 44.96 (113)	0.53
HLA-B*51:01	97.17 ± 43.31 (12)	107.76 ± 45.14 (106)	0.41
HLA-C*07:01	100.28 ± 37.53 (40)	109.97 ± 48.13 (78)	0.36
HLA-C*14:02	102.50 ± 53.63 (4)	106.83 ± 44.83 (114)	0.63
HLA-C*16:02	148.50 ± 111.02 (2)	105.97 ± 43.73 (116)	0.93
HLA-C*17:01	78.83 ± 18.97 (6)	108.18 ± 45.43 (112)	0.08
HLA-DQB1*03:01	112.09 ± 49.40 (54)	102.12 ± 40.55 (64)	0.33
HLA-DRB1*15:02	114.25 ± 49.00 (4)	106.42 ± 44.96 (114)	0.62

* values statistically significant.

**Table 4:** Associations between TC and LDL levels and the presence of non-GO protective alleles.

HLA	Allele (+)	Allele (-)	*p* Value
	Mean TC ± SD (*n*)	Mean TC ± SD (*n*)	
HLA-A*32:01	197.40 ± 70.69 (5)	177.88 ± 52.52 (113)	0.60
HLA-B*07:02	193.86 ± 62.66 (14)	176.67 ± 51.77 (104)	0.31
HLA-C*07:02	166.60 ± 61.73 (10)	179.83 ± 52.49 (108)	0.36
	**Mean LDL ± SD (*n*)**	**Mean LDL ± SD (*n*)**	
HLA-A*32:01	116.80 ± 51.46 (5)	106.24 ± 44.79 (113)	0.57
HLA-B*07:02	123.29 ± 55.97 (14)	104.45 ± 43.03 (104)	0.21
HLA-C*07:02	94.60 ± 49.27 (10)	107.81 ± 44.55 (108)	0.27

## Data Availability

The source data are available on reasonable request from the corresponding author.

## Abbreviations

AITD	autoimmune thyroid disorder
ApoA	apolipoprotein A
ApoB	apolipoprotein B
CAS	clinical activity score
DM1	diabetes mellitus type 1
EDTA	Ethylenediaminetetraacetic acid (anticoagulant)
GD	Graves’ disease
GO	Graves’ orbitopathy
GOHR	GO high risk alleles
GOP	GO protective alleles
HbA1c	glycosylated hemoglobin
HLA	human leukocyte antigens
IGF-1R	insulin-like growth factor 1 receptor
LDL	low-density lipoprotein
LDLR	LDL receptor
LPL	lipoprotein lipase
MHC	major histocompatibility complex
NGS	next-generation sequencing
non-GOHR	non-GO high risk alleles
non-GOP	alleles protective against non-GO
PCSK9	proprotein convertase subtilisin/kexin type 9
QoL	quality of life
RA	rheumatoid arthritis
SNPs	Single Nucleotide Polymorphism
TC	total cholesterol
TRAb	TSH-receptor antibodies
TSH	thyroid stimulating hormone (thyrotropin)
US	ultrasound

## References

[1] Zawadzka-Starczewska K., Tymoniuk B., Stasiak B., Lewiński A., Stasiak M. (2022). Actual Associations between HLA Haplotype and Graves’ Disease Development. J. Clin. Med..

[2] Ross D.S., Burch H.B., Cooper D.S., Greenlee M.C., Laurberg P., Maia A.L., Rivkees S.A., Samuels M., Sosa J.A., Stan M.N. (2016). 2016 American Thyroid Association Guidelines for diagnosis and management of hyperthyroidism and other causes of thyrotoxicosis. Thyroid.

[3] Bartalena L., Tanda M.L. (2022). Current concepts regarding Graves’ orbitopathy. J. Intern. Med..

[4] Krieger C.C., Morgan S.J., Neumann S., Gershengorn M.C. (2018). Thyroid stimulating hormone (TSH)/insulin-like growth factor 1 (IGF1) receptor cross-talk in human cells. Curr. Opin. Endocr. Metab. Res..

[5] Bartalena L., Kahaly G.J., Baldeschi L., Dayan C.M., Eckstein A., Marcocci C., Marinò M., Vaidya B., Wiersinga W.M., Ayvaz G. (2021). The 2021 European Group on Graves’ orbitopathy (EUGOGO) clinical practice guidelines for the medical management of Graves’ orbitopathy. Eur. J. Endocrinol..

[6] Stasiak M., Zawadzka-Starczewska K., Tymoniuk B., Stasiak B., Lewiński A. (2023). Significance of HLA in the development of Graves’ orbitopathy. Genes Immun..

[7] Ye X.Z., Huang S.S., Liu J., Lu B., Shao J.Q. (2019). High serum cholesterol: A novel risk factor for thyroid associated ophthalmopathy?. Zhonghua Nei Ke Za Zhi.

[8] Lanzolla G., Sabini E., Profilo M.A., Mazzi B., Sframeli A., Rocchi R., Menconi F., Leo M., Nardi M., Vitti P. (2018). Relationship between serum cholesterol and Graves’ orbitopathy (GO): A confirmatory study. J. Endocrinol. Investig..

[9] Zawadzka-Starczewska K., Stasiak B., Wojciechowska-Durczyńska K., Lewiński A., Stasiak M. (2022). Novel Insight into Non-Genetic Risk Factors of Graves’ Orbitopathy. Int. J. Environ. Res. Public Health.

[10] Di Taranto M.D., Fortunato G. (2023). Genetic Heterogeneity of Familial Hypercholesterolemia: Repercussions for Molecular Diagnosis. Int. J. Mol. Sci..

[11] Wong S.K., Ramli F.F., Ali A., Ibrahim N. (2022). Genetics of Cholesterol-Related Genes in Metabolic Syndrome: A Review of Current Evidence. Biomedicines.

[12] Mayor N.P., Hayhurst J.D., Turner T.R., Szydlo R.M., Shaw B.E., Bultitude W.P. (2019). Recipients Receiving Better HLA-Matched Hematopoietic Cell Transplantation Grafts, Uncovered by a Novel HLA Typing Method, Have Superior Survival: A Retrospective Study. Biol. Blood Marrow Transpl..

[13] MIA FORA Automation User Guideline. https://1drv.ms/b/s!Aiz1Ha7LrenIbNMnpeLaMuPbSDo?e=Edt5Db.

[14] Inoue D., Sato K., Enomoto T., Sugawa H., Maeda M., Inoko H. (1992). Correlation of HLA types and clinical findings in Japanese patients with hyperthyroid Graves’ disease: Evidence indicating the existence of four subpopulations. Clin. Endocrinol..

[15] Inoue D., Sato K., Maeda M., Inoko H., Tsuji K., Mori T. (1991). Genetic differences shown by HLA typing among Japanese patients with euthyroid Graves’ ophthalmopathy, Graves’ disease and Hashimoto’s thyroiditis: Genetic characteristics of euthyroid Graves’ ophthalmopathy. Clin. Endocrinol..

[16] Ohtsuka K., Nakamura Y. (1998). Human leukocyte antigens associated with hyperthyroid Graves ophthalmology in Japanese patients. Am. J. Ophthalmol..

[17] Mehraji Z., Farazmand A., Esteghamati A., Noshad S., Sadr M., Amirzargar S. (2017). Association of Human Leukocyte Antigens Class I and II with Graves’ Disease in Iranian Population. Iran J. Immunol..

[18] Huang X., Liu G., Mei S., Cai J., Rao J., Tang M. (2021). Human leucocyte antigen alleles confer susceptibility and progression to Graves’ ophthalmopathy in a Southern Chinese population. Br. J. Ophthalmol..

[19] Sabini E., Mazzi B., Profilo M.A., Mautone T., Casini G., Rocchi R., Ionni I., Menconi F., Leo M., Nardi M. (2018). High serum cholesterol is a novel risk factor for Graves’ orbitopathy: Results of a cross-sectional study. Thyroid.

[20] Ishina I.A., Zakharova M.Y., Kurbatskaia I.N., Mamedov A.E., Belogurov A.A., Gabibov A.G. (2023). MHC Class II Presentation in Autoimmunity. Cells.

[21] Terziroli Beretta-Piccoli B., Mieli-Vergani G., Vergani D. (2022). HLA, gut microbiome and hepatic autoimmunity. Front. Immunol..

[22] Miglioranza Scavuzzi B., van Drongelen V., Kaur B., Fox J.C., Liu J., Mesquita-Ferrari R.A., Kahlenberg J.M., Farkash E.A., Benavides F., Miller F.W. (2022). The lupus susceptibility allele DRB1*03:01 encodes a disease-driving epitope. Commun. Biol..

[23] Hunt P.J., Marshall S.E., Weetman A.P., Bunce M., Bell J.I., Wass J.A., Welsh K.I. (2001). Histocompatibility leucocyte antigens and closely linked immunomodulatory genes in autoimmune thyroid disease. Clin. Endocrinol..

[24] DR/DQ Associations. http://www.ctht.info/Table%2013%20DRB1%20DQA1%20DQB1%20associations%20in%20various%20populations.pdf.

[25] Common Associations of HLA-C alleles with Alleles of HLA-B. http://www.ctht.info/Table%209%20CB%20ASSOCIATIONS.pdf.

[26] Common Associations of HLA-B alleles with Alleles of HLA-C. http://www.ctht.info/Table%208%20BC%20ASSOCIATIONS.pdf.

[27] Takahasi K.R., Innan H. (2008). The direction of linkage disequilibrium: A new measure based on the ancestral-derived status of segregating alleles. Genetics.

[28] Vita R., Lapa D., Trimarchi F., Vita G., Fallahi P., Antonelli A., Benvenga S. (2017). Certain HLA alleles are associated with stress-triggered Graves’ disease and influence its course. Endocrine.

[29] Yanagawa T., Mangklabruks A., Chang Y.B., Okamoto Y., Fisfalen M.E., Curran P.G., DeGroot L.J. (1993). Human histocompatibility leukocyte antigen-DQA1*0501 allele associated with genetic susceptibility to Graves’ disease in a Caucasian population. J. Clin. Endocrinol. Metab..

[30] Odermarsky M., Nilsson A., Lernmark A., Sjöblad S., Liuba P. (2007). Atherogenic vascular and lipid phenotypes in young patients with Type 1 diabetes are associated with diabetes high-risk HLA genotype. Am. J. Physiol. Heart Circ. Physiol..

[31] Holoshitz J. (2010). The rheumatoid arthritis HLA-DRB1 shared epitope. Curr. Opin. Rheumatol..

[32] Toms T.E., Panoulas V.F., Smith J.P., Douglas K.M., Metsios G.S., Stavropoulos-Kalinoglou A., Kitas G.D. (2011). Rheumatoid arthritis susceptibility genes associate with lipid levels in patients with rheumatoid arthritis. Ann. Rheum. Dis..

[33] Egeberg A., Hansen P.R., Gislason G.H., Thyssen J.P. (2016). Clustering of autoimmune diseases in patients with rosacea. J. Am. Acad. Dermatol..

[34] Xiao W., Li J., Huang X., Zhu Q., Liu T., Xie H., Deng Z., Tang Y. (2022). Mediation roles of neutrophils and high-density lipoprotein (HDL) on the relationship between HLA-DQB1 and rosacea. Ann. Med..

[35] Yang F., Sun L., Zhu X., Han J., Zeng Y., Nie C., Yuan H., Li X., Shi X., Yang Y. (2017). Identification of new genetic variants of HLA-DQB1 associated with human longevity and lipid homeostasis-a cross-sectional study in a Chinese population. Aging.

[36] Mostafazadeh A., Saravi M., Niaki H.A., Drabbels J., Gholipour H.M., Minagar M., Mosavi E., Jalali F., Bijani A. (2011). HLA-DRBeta1, circulating Th1/Th2 cytokines and immunological homunculus in coronary atherosclerosis. Iran J. Allergy Asthma Immunol..

